# Real-Time Cascaded State Estimation Framework on Lie Groups for Legged Robots Using Proprioception

**DOI:** 10.3390/biomimetics10080527

**Published:** 2025-08-12

**Authors:** Botao Liu, Fei Meng, Zhihao Zhang, Maosen Wang, Tianqi Wang, Xuechao Chen, Zhangguo Yu

**Affiliations:** School of Mechatronical Engineering, Beijing Institute of Technology, Beijing 100811, China; lbt0116@bit.edu.cn (B.L.); zhihao_zhang@bit.edu.cn (Z.Z.); wangmaosen@bit.edu.cn (M.W.); wangtianqi_real@bit.edu.cn (T.W.); chenxuechao@bit.edu.cn (X.C.); yuzg@bit.edu.cn (Z.Y.)

**Keywords:** state estimation, proprioception, Kalman filter, moving horizon estimation

## Abstract

This paper proposes a cascaded state estimation framework based on proprioception for robots. A generalized-momentum-based Kalman filter (GMKF) estimates the ground reaction forces at the feet through joint torques, which are then input into an error-state Kalman filter (ESKF) to obtain the robot’s prior state estimate. The system’s dynamic equations on the Lie group are parameterized using canonical coordinates of the first kind, and variations in the tangent space are mapped to the Lie algebra via the inverse of the right trivialization. The resulting parameterized system state equations, combined with the prior estimates and a sliding window, are formulated as a moving horizon estimation (MHE) problem, which is ultimately solved using a parallel real-time iteration (Para-RTI) technique. The proposed framework operates on manifolds, providing a tightly coupled estimation with higher accuracy and real-time performance, and is better suited to handle the impact noise during foot–ground contact in legged robots. Experiments were conducted on the BQR3 robot, and comparisons with measurements from a Vicon motion capture system validate the superiority and effectiveness of the proposed method.

## 1. Introduction

Legged robots have witnessed rapid advancements in recent years. Compared to traditional wheeled and tracked robots, their superior mobility and obstacle-negotiation capabilities exhibit significant potential across various task scenarios such as patrolling, search and rescue, and transportation [1,2,3]. State estimation plays a crucial role in the operations of legged robots. Accurate state estimation not only provides precise coordinates within the global frame but also supplies smooth and accurate signals to the controller. However, because legged robots are complex electromechanical systems, their variable working environments, continuous multi-support movement characteristics, underactuation, and nonlinear system properties pose considerable challenges to state estimation.

Various methods have been applied to the state estimation problem in legged robots. The Extended Kalman Filter (EKF) has been introduced for its lightweight nature and robustness; for instance, Sola et al. developed a quaternion-based QEKF [4]. Hartley, taking into account the right-invariant properties on Lie groups, developed a contact-aided invariant Extended Kalman Filter (IEKF) [5]. Li further incorporated dynamic characteristics into this framework, enhancing the estimator’s adaptability to contact slippage [6]. However, Kalman filter-based methods can only handle Gaussian noise and cannot adequately address constraints. Consequently, optimization-based tightly coupled methods have been introduced. Wisth et al. developed a state estimation framework based on factor graphs, which is often used for processing information from external sensors such as vision [7]. Although integrating multiple sensors brings excellent estimation performance, it also incurs high computational costs, posing challenges to real-time applications [8,9]. In addition, external sensors may be unstable and are prone to being affected by the environment. For example, under adverse weather conditions such as heavy rain, thick fog, and heavy snow, the accuracy of optical sensors will decrease because the propagation of light is hindered, resulting in blurred images or data being obtained. Therefore, proprioceptive estimation remains necessary [10].

Moving horizon estimation is the dual problem of Model Predictive Control (MPC) [11]. It integrates the system model over a finite past time horizon and fuses all observational information within this horizon to obtain the optimal state estimate sequence [12,13]. This method can handle non-Gaussian noise and constraints, making it well-suited for legged robots with frequent contact switches, thereby reducing noise impact at the moments of ground contact. While MHE theory has been extensively studied, its application to legged robots remains limited. For example, Khorshidi combined Dynamic Mode Decomposition (DMD) with MHE to estimate the centroidal states of a quadruped robot, but this method relies on neural networks for estimating centroidal dynamics, making it challenging to apply at high update frequencies on real hardware [14]. Kang proposed a distributed state estimation method combining EKF and MHE, where the nonlinear parts are separately estimated. While this method is fast, it does not fully utilize the advantages of MHE [15].

In previous studies, Euler angles and quaternions were commonly used to describe rigid body motion [4,10]. However, Euler angles suffer from gimbal lock, and quaternions have the issue of double coverage. To overcome these limitations, Lie group representations have been introduced [16,17]. Nevertheless, the over-parameterization of rotation matrices makes it challenging to directly construct the state equations. In optimal control, error dynamics based on variational methods are often employed [18,19], but extending these methods to MHE is difficult. This paper employs canonical coordinates of the first kind on the Special Euclidean Group (SE(3)) to parameterize rigid body motion [20], which naturally avoids tensor usage while preserving structural properties. The Cayley transform, as a third-order approximation of the exponential map, ensures both computational efficiency and sufficient accuracy.

MHE, being an optimization-based tightly coupled method, relies heavily on solving speed and update frequency for its performance. In nonlinear Model Predictive Control (NMPC), methods such as sequential quadratic programming (SQP) [21,22] and real-time iteration (RTI) [23,24,25] have been widely explored for real-time optimization, and these methods can easily be extended to MHE. RTI, known for its strong real-time capability, has already been shown to offer optimality under certain conditions.

The main contribution of this paper is the introduction of a proprioceptive tightly coupled state estimation framework based on Lie group representations. The framework first uses an ESKF to provide prior estimates of the robot’s state, followed by MHE to obtain the final state estimation. Our ESKF incorporates joint torque information, which not only improves the estimation accuracy compared to existing methods but also allows for the estimation of external forces acting on the robot. MHE is formulated using canonical coordinates of the first kind based on the Cayley transform in the right-trivialized tangent space of SE(3), combining past observations and prior information from the ESKF to achieve tightly coupled estimation. To further reduce the delay caused by optimization, we propose a parallel RTI method, enabling high-speed state updates. Finally, experiments conducted on the BQR3 robot validate the effectiveness and superiority of the proposed method.

The paper is structured as follows. The system modeling on Lie group is described in Section 2. The prior estimation method of ESKF incorporating torque information is presented in Section 3. Section 4 discusses the implementation of the tightly coupled MHE framework and the Para-RTI solution method. Furthermore, Section 5 conducts experimental validation of the proposed framework, and finally the paper is concluded in Section 6.

## 2. System Modeling Using Canonical Coordinates of the First Kind

### 2.1. Cayley Map and Right-Trivialized Tangent

For dynamical systems, their configuration space can be represented on a Lie group *G*, with the corresponding tangent space referred to as the Lie algebra g. The Lie algebra can be mapped from the vector space $R^{n}$ using the hat operator $\hat{\left(\cdot \right)}:R^{n}\to g$, and the inverse mapping is represented by the vee map ${\left(\cdot \right)}^{\vee }:g\to R^{n}$. Retraction maps φ:g→G are used to relate the Lie group to its corresponding Lie algebra and can be written as

(1) $$g=\varphi (\hat{\rho })$$

where g∈G and $\rho \in R^{6}$.

The exponential map exp can be used as a kind of retraction map, which is also called canonical coordinates of the first kind. The exponential map provides an exact representation of rigid body motion on a Lie group. However, due to its computational complexity, this paper considers a third-order approximation $\mathrm{cay}\left(\hat{\rho }\right)=\exp\left(\hat{\rho }\right)+O\left(∥\hat{\rho }{∥}^{3}\right)$ (which is only valid for the rigid motion groups like the Special Orthogonal Group (SO(3)) and SE(3)), and the Cayley map is defined as follows:

(2) $$g=\mathrm{cay}\left(\hat{\rho }\right)={\left(I-\hat{\rho }/2\right)}^{-1}\left(I+\hat{\rho }/2\right)$$

(3) $$\hat{\rho }={\mathrm{cay}}^{-1}\left(g\right)=2\left(g-I\right){\left(g+I\right)}^{-1}$$

where I is the identity matrix.

The Cayley map does not involve transcendental functions and is easier to differentiate. For the twist $\xi \in R^{n}$ on the group, it has the Poisson relation $\dot{g}=g\hat{\xi }$ which is a differential equation. To solve the differential equation, use right-trivialized tangent $d\varphi_{\rho }:g\to T_{g}G$ (Tangent space on *g*):

(4) $$\begin{array}{cc} \hat{\xi } & =g^{-1}\dot{g}\\ \; & =g^{-1}\frac{d}{dt}\mathrm{cay}\left(\hat{\rho }\right)\\ \; & =g^{-1}\mathrm{cay}\left(\hat{\rho }\right){\mathrm{dcay}}_{\rho }\left(\dot{\hat{\rho }}\right)\\ \; & ={\mathrm{cay}}^{-1}\left(\hat{\rho }\right)\mathrm{cay}\left(\hat{\rho }\right){\mathrm{dcay}}_{\rho }\left(\dot{\hat{\rho }}\right)\\ \; & ={\mathrm{dcay}}_{\rho }\left(\dot{\hat{\rho }}\right) \end{array}$$

where $\frac{d}{dt}\mathrm{cay}\left(\hat{\rho }\right)=\mathrm{cay}\left(\hat{\rho }\right){\mathrm{dcay}}_{\rho }\left(\dot{\hat{\rho }}\right)$ is derived from the chain rule.

Then the local reconstruction equation can be obtained from the inverse of Equation (4):

(5) $$\dot{\hat{\rho }}={\mathrm{dcay}}_{\rho }^{-1}\left(\hat{\xi }\right)$$

**Figure 1:**
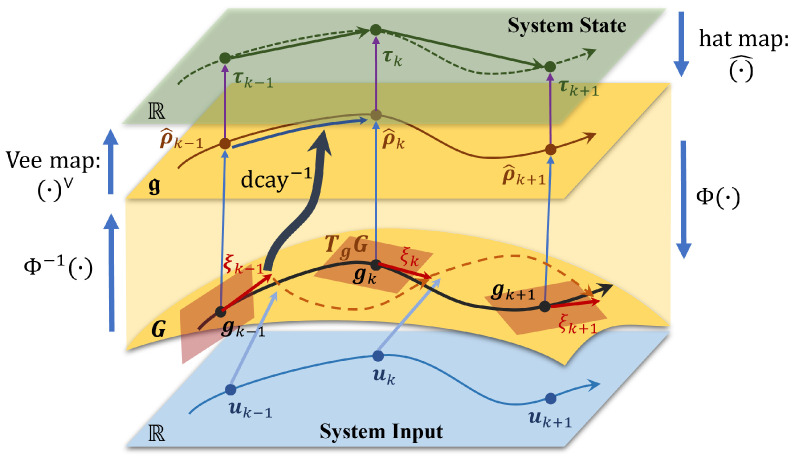
Illustration of the system model on manifold. The system model evolves on the manifold SE(3), denoted as *G*. The rigid body motion is mapped to the Lie algebra g through the Cayley map. ${\mathrm{dcay}}^{-1}$ maps the variations in the tangent space of the Lie group back to the Lie algebra, thereby parameterizing the system dynamics, which evolve on the Lie algebra. The parameterized system state is then mapped to Euclidean space via the vee map.

The system model and mapping relationships on the manifold are illustrated in Figure 1.

### 2.2. Configuration of Rigid Body on Lie Groups

In our case, the rigid body motion evolves on the Special Euclidean group SE(3), which represents the set of all rigid body transformations in three-dimensional space. It is a Lie group that combines rotation and translation, and it can be expressed as

(6) $$T=\left[\begin{array}{cc} r & p\\ 0 & 1 \end{array}\right]\in SE\left(3\right)$$

where R is a rotation matrix in the Special Orthogonal Group SO(3), and $p\in R^{3}$ is a translation vector. The tangent vector field at the identity element of a Lie group *G* is defined as the Lie algebra g.

For T∈SE(3), the corresponding Lie algebra can be expressed in the following form:

(7) $$\hat{\rho }=\left[\begin{array}{cc} \theta^{\times } & r\\ 0 & 0 \end{array}\right]\in \mathrm{se}\left(3\right),\;\theta^{\times }=\left[\begin{array}{ccc} 0 & -\theta_{3} & \theta_{2}\\ \theta_{3} & 0 & -\theta_{1}\\ -\theta_{2} & \theta_{1} & 0 \end{array}\right]$$

where $\theta \in R^{3}$ and $r\in R^{3}$, while the operator ${\left(\cdot \right)}^{\times }:R^{3}\to \mathrm{so}\left(3\right)$ denotes the skew-symmetric matrix that maps from the Lie algebra to the vector space.

Because se(3) is obviously diffeomorphic to $R^{6}$, we can get the parameterized equation from Equation (5):

(8) $$\dot{\rho }={\mathrm{dcay}}_{\rho }^{-1}\left(\xi \right),\rho =\left[\begin{array}{c} \theta \\ r \end{array}\right]\in R^{6},\xi =\left[\begin{array}{c} \omega \\ v \end{array}\right]\in R^{6}$$

where $\theta \in R^{3}$ and $r\in R^{3}$ are angular and linear velocity, respectively, and the inverse Cayley tangent can be derived simply as [20]

(9) $${\mathrm{dcay}}_{\left(\theta ,r\right)}^{-1}=\left[\begin{array}{cc} I_{3}-\frac{1}{2}\theta^{\times }+\frac{1}{4}\theta \theta^{T} & 0_{3}\\ -\frac{1}{2}\left(I_{3}-\frac{1}{2}\theta^{\times }\right)r^{\times } & I_{3}-\frac{1}{2}\theta^{\times } \end{array}\right]$$

The dynamic equation of a rigid body on SE(3) can be described by the Euler–Poincaré equation:

(10) $$\dot{\xi }=J_{b}^{-1}\left(-{\mathrm{ad}}_{\xi }^{*}J_{b}\xi +u\right)$$

where $J_{b}$ is the mass–inertia matrix composed by inertia matrix I and mass *m*

(11) $$J_{b}=\left[\begin{array}{cc} I_{b} & 0\\ 0 & mI_{3} \end{array}\right]\in R^{6\times 6}$$

u is the system input and ${\mathrm{ad}}_{\xi }$ is called the adjoint operator and is given by

(12) $${\mathrm{ad}}_{\xi }^{*}=-{\mathrm{ad}}_{\xi }^{T}=\left[\begin{array}{cc} -\omega^{\times } & -v^{\times }\\ 0 & -\omega^{\times } \end{array}\right]$$

Combining Equation (8) and Equation (10), we can obtain the complete system equation of state:

(13) $$\dot{x}=\left[\begin{array}{c} \dot{\rho }\\ \dot{\xi } \end{array}\right]=\underset{f(x,u)}{\underset{︸}{\left[\begin{array}{c} {\mathrm{dcay}}_{\rho }^{-1}\left(\xi \right)\\ J_{b}^{-1}\left(-{\mathrm{ad}}_{\xi }^{*}J_{b}\xi +u\right) \end{array}\right]}}$$

Optimization problems for nonlinear models are often solved by linearizing the system state equations to the first order as follows:

(14) $$\dot{x}=Ax+Bu$$

where

(15) $$\begin{array}{cc} A & =\frac{\partial f(x,u)}{\partial x}=\left[\begin{array}{cc} \frac{\partial \left({\mathrm{dcay}}_{\rho }^{-1}\xi \right)}{\partial \rho } & {\mathrm{dcay}}_{\rho }^{-1}\\ 0 & J_{b}^{-1}\frac{\partial \left(Ad_{\xi }^{T}J_{b}\xi \right)}{\partial \xi } \end{array}\right]\\ B & =\frac{\partial f(x,u)}{\partial u}=\left[\begin{array}{c} 0\\ J_{b}^{-1} \end{array}\right] \end{array}$$

## 3. Error-State Kalman Filter with Joint Torque

In the previous section, we derived the state equations of the system. However, when modeling actual dynamical systems, it is essential to account for additional factors such as system noise. Within the framework proposed in this paper, it is necessary to model not only the basic state information but also the sensor noise and biases. Moreover, the identification of inputs to the dynamical system becomes crucial with the incorporation of dynamics. This section presents an estimator based on the ESKF to estimate the biases of the robot’s sensors and the external wrench acting on it.

### 3.1. GRF Reconstruction

Dynamical systems are typically complex and nonlinear, making it crucial to accurately account for system dynamics to achieve precise state estimation. In this paper, we simplify the dynamical system of a legged robot to a single rigid body model. The input u is a six-dimensional force vector ${\left[M_{x},M_{y},M_{z},F_{x},F_{y},F_{z}\right]}^{T}$ acting on the torso, which generally consists of contact forces and gravitational forces. Contact forces arise from interactions with the environment, including the ground reaction forces (GRF) $f_{c}={\left[f_{cx},f_{cy},f_{cz}\right]}^{T}$ at the foot and external interaction wrench $u_{e}={\left[M_{ex},M_{ey},M_{ez},F_{ex},F_{ey},F_{ez}\right]}^{T}$. Therefore, u in Equation (14) can be rewritten as

(16) $$u=\sum_{i=1}^{n}\left[\begin{array}{c} r_{i}^{\times }\\ I \end{array}\right]f_{c,i}+u_{e}+\left[\begin{array}{c} 0\\ mg \end{array}\right]$$

where $g={\left[0,0,-9.8\right]}^{T}$ is the gravity vector and *n* is the number of stance legs.

The reconstruction of GRF can be achieved through a Kalman filter using the generalized momentum equation for a single leg, which can be expressed as

(17) $$\dot{h}-C{\left(q,\dot{q}\right)}^{T}\dot{q}+G\left(q\right)=\tau +J_{c}^{T}f_{c}$$

where $h=M\left(q\right)\dot{q}$ is the generalized momentum, and M(q), $C(q,\dot{q})$, and g(q) are the inertia matrix, Coriolis matrix, and gravity matrix, respectively. q and $\dot{q}$ are the joint position and joint velocity. τ is the vector of the actuated joint torques, and $J_{c}$ is the corresponding Jacobian.

The GRF can be modeled as random walk process $\dot{f_{c}}=n_{f}$ and $n_{f}$ is the Gaussian noise. The leg model can be obtained as

(18) $$\left[\begin{array}{c} \dot{h}\\ \dot{f_{c}} \end{array}\right]=\underset{A_{c}}{\underset{︸}{\left[\begin{array}{cc} 0 & J_{c}^{T}\\ 0 & 0 \end{array}\right]}}\left[\begin{array}{c} h\\ f_{c} \end{array}\right]+\underset{B_{c}}{\underset{︸}{\left[\begin{array}{c} I\\ 0 \end{array}\right]}}u+n_{h}$$

where $u=C{\left(q,\dot{q}\right)}^{T}\dot{q}-G\left(q\right)+\tau$ includes all the nonlinear dynamics.

As the generalized momentum h can be measured from joint encoders and with the high transparency joint, the GRF measurement can be obtained from joint torque. The measurement model can be defined as

(19) $$\underset{z}{\underset{︸}{\left[\begin{array}{c} h_{m}\\ f_{m} \end{array}\right]}}=Hx+n_{m}$$

where H is an identity matrix and $n_{m}$ is the Gaussian noise.

With Equations (18) and (19), the GRF $f_{c}$ can be obtained from a standard Kalman filter.

In practical applications, most mainstream legged robots today are equipped with high-performance planetary gearboxes, typically with a reduction ratio below 30. Extensive experimental evidence has shown that such gearboxes, when operating at low reduction ratios, can effectively reduce gearbox friction and non-ideal actuator effects, thus achieving high torque control transparency. Therefore, in the process of joint torque estimation and GRF reconstruction, it is reasonable to approximate that the output joint torque closely corresponds to the actual force applied.

### 3.2. Error-State Kalman Filter

Consider the equations of motion of a rigid body on the SE(3) group, which, unlike the modeling in vector space in the previous section, can be written as

(20) $$\begin{array}{cc} \dot{g} & =g\hat{\xi }\\ \dot{\xi } & =J_{b}^{-1}\left(-{\mathrm{ad}}_{\xi }^{*}J_{b}\xi +u\right) \end{array}$$

To incorporate the biases of the accelerometer and gyroscope in the IMU, as well as the external wrench acting on the system, the true state variables of the system can be expanded as

(21) $$s:=\left(g,\xi ,B,u_{e}\right)$$

By modeling the biases and external wrench as random walk processes, the system’s true state equations can be formulated to reflect these stochastic components as follows:

(22) $$\begin{array}{cc} \dot{g} & =g\hat{\xi }\\ \dot{\xi } & =J_{b}^{-1}\left(-{\mathrm{ad}}_{\xi }^{*}J_{b}\xi +u_{c}+u_{e}+u_{g}\right)+n_{uc}\\ \dot{b} & =n_{b}\\ {\dot{u}}_{e} & =n_{ue} \end{array}$$

where $n_{uc}$, $n_{b}$, and $n_{ue}$ are the corresponding Gaussian noise.

Considering that the prediction in Kalman filter occurs only within a single time step, we approximate the infinitesimal transformation on the Lie group to linearize the system state equations:

(23) $$\delta g={\left.\frac{d}{d\epsilon }\right|}_{\epsilon =0}g\exp\left(\epsilon \delta \hat{\zeta }\right)=g\delta \hat{\zeta }$$

Using the feature of skew-symmetric matrix, $a^{\times }b=a\times b=-b\times a=-b^{\times }a,\forall a,b\in R^{3}$, the error-state model can be derived as

(24) $$\left[\begin{array}{c} \delta \dot{\zeta }\\ \delta \dot{\xi }\\ \delta \dot{b}\\ \delta {\dot{u}}_{e} \end{array}\right]=\left[\begin{array}{cccc} -{\mathrm{ad}}_{\xi } & I & 0 & 0\\ 0 & J_{b}^{-1}\left(C-{\mathrm{ad}}_{\xi }^{*}J_{b}\right) & 0 & J_{b}^{-1}\\ 0 & 0 & 0 & 0\\ 0 & 0 & 0 & 0 \end{array}\right]\left[\begin{array}{c} \delta \zeta \\ \delta \xi \\ \delta b\\ \delta u_{e} \end{array}\right]+\left[\begin{array}{c} 0\\ n_{uc}\\ n_{b}\\ n_{ue} \end{array}\right]$$

where

(25) $$C=\left[\begin{array}{cc} {\left(I_{b}\omega \right)}^{\times } & mv^{\times }\\ mv^{\times } & 0 \end{array}\right]$$

Equation (24) can be easily rewritten to the discrete linearized form by using the forward Euler method. Then the error-state estimation is

(26) $$\delta s_{k}=K_{k}\left(z_{k}-h\left(s_{k}\right)\right)$$

where

(27) $$z_{k}-h\left(s_{k}\right)=\left[\begin{array}{c} a_{m}-\frac{u_{c}+u_{g}+u_{e}}{m}+b_{a}\\ \omega_{m}-\omega +b_{g}\\ v_{m}-v\\ \log{\left(g^{-1}g_{m}\right)}^{\vee } \end{array}\right]$$

Then the measurement matrix $H_{k}$ can be derived by using the chain rule. The implementation details of the ESKF are given in Algorithm 1.
**Algorithm 1** Error-state Kalman filter with joint torque.Initialize the state variable and covariance matrix${\tilde{s}}_{0}=0$, ${\tilde{P}}_{0}=I$**repeat**   Compute state   $\delta s_{k}=A_{e,k}\delta {\tilde{s}}_{k-1}$   Compute covariance estimation   $P_{k}=A_{e,k}{\tilde{P}}_{k-1}A_{e,k}^{T}+Q_{k}$   Compute optimal Kalman gain   $K_{k}=P_{k}H_{k}^{T}{\left(H_{k}P_{k}H_{k}^{T}+R_{k}\right)}^{-1}$   Update state estimation with measurement   $\delta {\tilde{s}}_{k}=K_{k}\left(z_{k}-h\left(s_{k}\right)\right)$   Update covariance estimation   ${\tilde{P}}_{k}=\left(I-K_{k}H_{k}\right)P_{k}$**until** stop

## 4. MHE with Para-RTI

**Figure 2:**
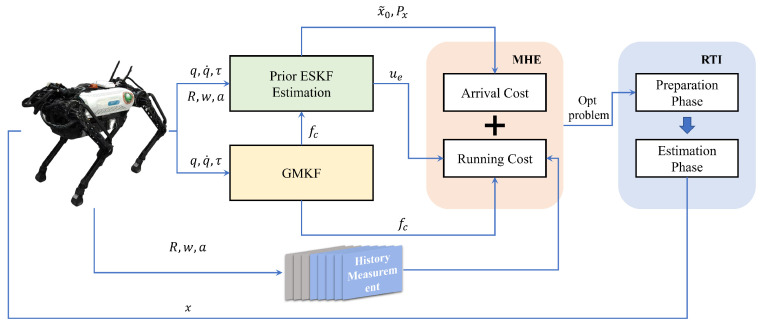
Cascaded state estimate framework. The joint torques from the robot are input into the first stage of the GMKF to compute the ground reaction forces at the feet. The resulting forces, along with kinematic data and IMU information, are then fed into the ESKF. The system’s prior state estimate, together with historical measurement data from the sliding window, is formulated as an MHE problem. Finally, the Para-RTI method is employed to solve the optimization problem, enabling high-speed updates of the state estimates.

To enable accurate and real-time state estimation for nonlinear systems under constraints, MHE has emerged as a powerful optimization-based framework. However, its practical deployment is often hindered by high computational demands, especially in fast-sampling or resource-limited scenarios. To address this issue, we adopt an efficient implementation of MHE based on the parallelizable real-time iteration (Para-RTI) scheme, which exploits parallel computation across the estimation horizon to significantly reduce latency while preserving estimation accuracy. The whole state estimation framework is shown in Figure 2.

### 4.1. MHE

Although Kalman filter is simple and effective, with the advancement of onboard computational power, state estimation problems are no longer satisfied with using only the Kalman filter. Optimization-based methods can better handle complex nonlinear systems, constraints, and non-Gaussian noise, and they also facilitate multi-sensor fusion. Optimization-based methods can be seen as solving the Maximum A Posteriori (MAP) problem, where MHE achieves state estimation by minimizing a cost function over a finite time window. MHE is a recursive implementation of MAP estimation and can be written as

(28a) $$\begin{array}{cc} \min_{\begin{array}{c} X\left[0:n\right] \end{array}}\;\; & \frac{1}{2}{∥x_{0}-{\tilde{x}}_{0}∥}_{Q}^{2}+\sum_{k=0}^{n-1}\frac{1}{2}{∥e_{x,k}∥}_{P_{x}}^{2}+\sum_{k=0}^{n}\frac{1}{2}{∥e_{y,k}∥}_{P_{y}}^{2} \end{array}$$

(28b) $$\begin{array}{cc} \;s.t.\; & x_{k+1}=f\left(x_{k},u_{k}\right)+e_{x,k} \end{array}$$

(28c) $$\begin{array}{cc} \; & y_{k}=h\left(x_{k},u_{k}\right)+e_{y,k} \end{array}$$

(28d) $$\begin{array}{cc} \; & g\left(x_{k},u_{k}\right)\leq 0,\;k=0,\ldots ,n \end{array}$$

where $X_{0:n}=\left[x_{0:n},e_{x,0:n},e_{y,0:n}\right]$ is a sequence that contains all state variables and error terms within the finite horizon, *n* is the number of time steps, Q, $P_{x}$, and $P_{y}$ are corresponding weight matrices. Equations (28b) and (28c) represent the state equation and observation equation as constraints, respectively, while Equation (28d) represents the inequality constraints. The first term of the cost function is typically referred to as the arrival cost. Here, ${\tilde{x}}_{0}$ is the prior estimate, which, in this paper, is updated using the state estimate from the ESKF designed in the previous section. The other terms are the running cost, which quantify the process error and measurement error.

Unlike the weighted matrices in MPC, which can be freely adjusted, the weighting matrices in MHE have a significant impact on the estimation results. They are typically inversely proportional to the noise covariance of the system. The noise covariance can be obtained from the Kalman filter to ensure that the final estimation result approximates the maximum likelihood estimate.

### 4.2. Para-RTI

Nonlinear models typically impose significant computational burdens on the solution of MHE. As the dual problem of MPC, the solution methodologies for NMPC have become relatively mature, and the efficacy of RTI techniques has been validated. In this paper, we extend the application of RTI to the solution of MHE. RTI can be viewed as a specialized application of SQP. By performing a first-order linearization of the nonlinear system model and compressing the problem into a quadratic programming (QP) subproblem, RTI executes a single Newton step at each sampling time. Each individual RTI step is divided into a preparation phase and a feedback phase.
**Algorithm 2** Para-RTI: Parallel real-time iteration for nonlinear MHE.1:**Initialization:** Initialize Kalman Filter (KF), Preparation Thread, and Estimation Thread2:**Main Thread:**3:**while** true **do**4:   Run Kalman Filters to obtain prior estimate ${\hat{x}}_{0}$, bias b, and external wrench $u_{e}$5:   Send KF results to Preparation Thread6:   Wait for sensor measurements $y_{k}$ and send sensor measurements to Estimation Thread7:   Receive estimated values from Estimation Thread8:   Update system state with estimated values9:**end while**10:**Preparation Thread:**11:**while** true **do**12:   Wait for prior results ${\tilde{x}}_{0}$ from Main Thread13:   Evaluate objective in Equation (28a), constraints in Equations (28b)–(28d), and sensitive matrices A,B in Equation (15)14:   Store evaluation results for Estimation Thread15:**end while**16:**Estimation Thread:**17:**while** true **do**18:   Wait for sensor measurements $y_{k}$ from Main Thread19:   Retrieve evaluation results from Preparation Thread20:   Solve QP problem using evaluation results21:   Send estimated values to Main Thread22:**end while**

During the preparation phase, which is executed before new measurement data is acquired, the system model is linearized, and the solution from the previous time step is used as the initial guess. The entire Nonlinear Programming (NLP) problem is then constructed into a QP subproblem. The feedback phase, which occurs immediately after new measurement data is available, involves solving the previously constructed QP problem. The last element in the resulting solution sequence is taken as the current state estimate to be sent to the controller.

**Figure 3:**
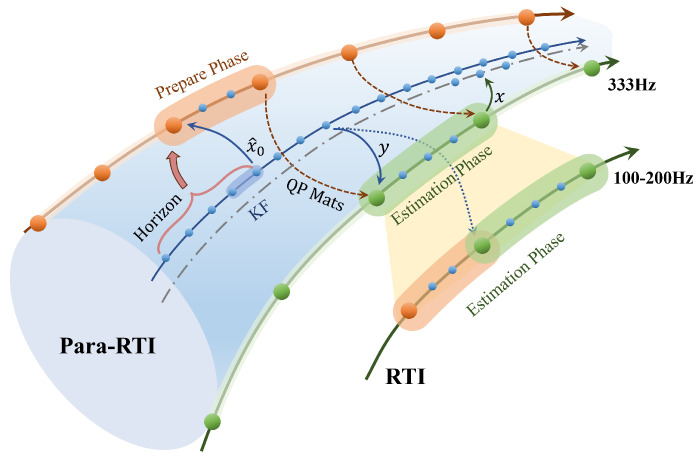
The working principle of Para-RTI and comparison with standard RTI.

To further accelerate the update frequency of MHE, we propose a Para-RTI method. Para-RTI is also divided into a preparation phase and an estimation phase. However, unlike standard RTI, the preparation and estimation phases are executed in two separate threads outside the main thread. A high-real-time Kalman Filter runs in the main thread, calculating the prior estimate, bias, and external wrench in each cycle. The preparation thread obtains the Kalman Filter results from the main thread in each cycle, and evaluates the objective, constraints, and corresponding sensitivities. The estimation thread then solves the QP problem using the results from the preparation thread each time sensor measurements are received from the main thread, and the estimated values are sent back to the main thread. The continuous execution of Para-RTI compresses the estimation time to the QP problem-solving time, thereby enhancing the estimation speed of NMHE. The structure of Para-RTI is shown in Figure 3.

In this paper, Equation (28b) adopts the linearized form presented in Equation (14), with the input u being computed within ESKF. Additionally, the observation equation incorporates the computed biases as

(29) $$\left[\begin{array}{c} {\mathrm{cay}}^{-1}{\left(g_{m,k}\right)}^{\vee }\\ \xi_{m,k}+b_{g,k} \end{array}\right]=\left[\begin{array}{c} \rho_{k}\\ \xi_{k} \end{array}\right]$$

Without inequality constraints, the QP subproblem has the standard form as

(30) $$\begin{array}{cc} \min\;\; & \frac{1}{2}X^{⊤}HX+h^{⊤}X\\ \;s.t.\;\;\; & CX=D. \end{array}$$

## 5. Experiments

Experiments were conducted on a quadruped robot named BQR3, which weighs 50 kg and measures 0.8 m in length and 0.5 m in height. It is equipped with 12 joints, each integrated with absolute encoders, and an MTi-300 IMU mounted at the center of the torso. Thanks to the high transmission transparency of the joints, joint torques can be computed in real time using actuator current feedback. The proposed framework was deployed on a mini PC (SER07) powered by an AMD Ryzen R7 7840HS CPU (P-core 5.10 GHz, E-core 3.80 GHz).

To evaluate estimation accuracy, a Vicon motion capture system was used to record the absolute position and orientation of the robot’s center of mass.

**Figure 4:**
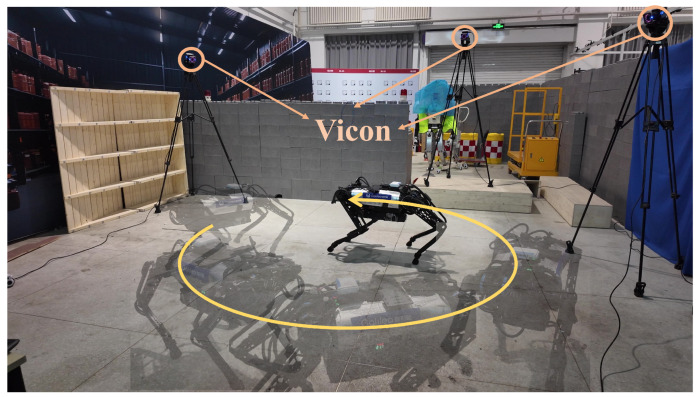
Experiment without disturbance snapshot. The robot operates within a 4 × 4 m area, and an external motion capture system composed of six Vicon cameras is used to obtain the robot’s position and orientation measurements.

**Figure 5:**
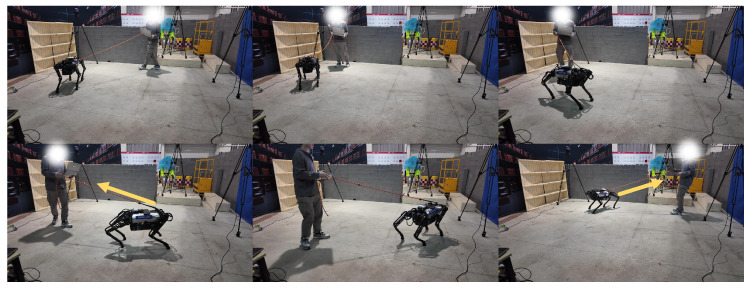
Experiment with disturbance snapshot.

Two sets of experiments were conducted: circular walking without disturbance (Figure 4) and with external disturbances (Figure 5). All experiments were carried out on a flat terrain within a confined area. The robot was teleoperated using velocity commands, including sudden stops and acceleration/deceleration. In disturbance experiments, random external perturbations were applied via a rope attached to the torso. The robot was controlled by an NMPC controller running at 200 Hz, the ESKF operated at 1000 Hz, and, enabled by the proposed parallel computing framework, and the MHE state estimator was updated at 333 Hz with a prediction horizon of 25 steps.

While many modern embedded platforms (such as those running Linux, RTOS, or equipped with multi-core processors) do support multi-threading, their computational resources and memory are usually much more limited compared to desktop systems. Multi-threading may introduce overhead from context switching and resource contention, and careful scheduling is needed to ensure that real-time performance is not compromised. For resource-constrained embedded platforms, it is often preferable to adopt state estimation methods with lower computational requirements, such as Kalman filter-based approaches, or to use the original RTI method instead of the parallelized Para-RTI scheme. These alternatives can help ensure reliable and timely state estimation without overloading the system.

**Figure 6:**
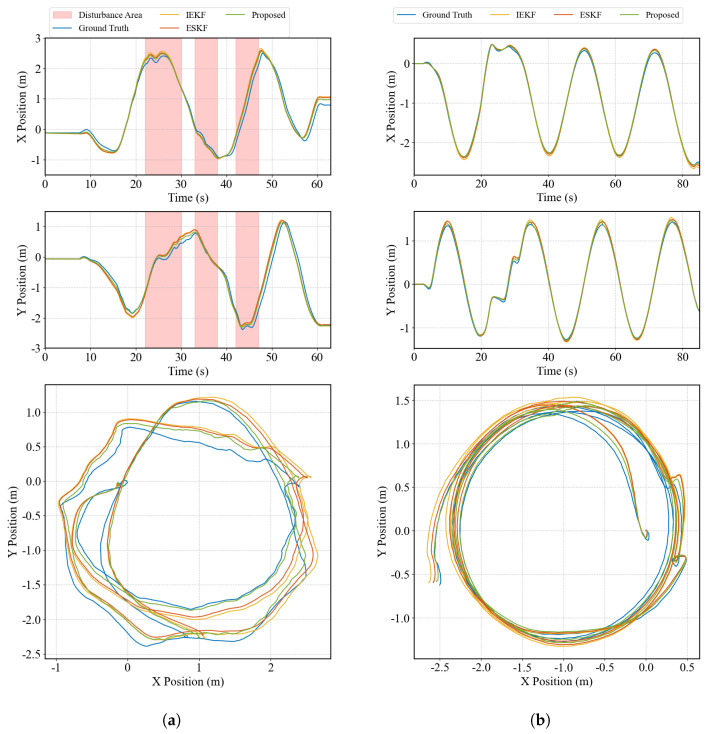
The comparison of absolute positions in the space. (**a**) shows results under undisturbed conditions, (**b**) presents the position estimation results under external disturbances.

To validate the superiority and effectiveness of the proposed method, comparisons were made not only with Vicon-based ground truth but also with ESKF and the IEKF method from [5]. Figure 6 presents the absolute position estimation results from different estimators under both experimental conditions. It is noteworthy that the ESKF output serves as prior input to the MHE, and the MHE weighting is set as the inverse of the ESKF noise covariance.

Figure 6a shows results under undisturbed conditions, including time-series data of the x and y positions and the 2D trajectory. The z-direction is not shown, as vertical drift is unacceptable for quadruped robots and is typically compensated using a weighted average of kinematic foot positions. The vertical position of the robot’s base is recalibrated at each step by computing a weighted average of the positions of the stance foot ends, ensuring that the base height is consistent with the ground contact constraints. The corresponding equation is as follows:

(31) $$z_{\mathrm{base}}=\frac{1}{n}\sum_{i=1}^{n}\cdot z_{\mathrm{foot},i}$$

where $w_{i}$ is the weight for the *i*-th stance foot (with $\sum w_{i}=1$), and $z_{\mathrm{foot},i}$ is the vertical position of the *i*-th stance foot end. This description has been integrated into the methodology for reproducibility.

Compared with the Vicon ground truth, the proposed method demonstrates superior tracking accuracy. ESKF and IEKF, due to their single-step optimal estimation nature, struggle to handle impact-induced noise during foot contact. Since the proposed state estimation framework relies entirely on proprioceptive sensors, drift is inevitable. Nevertheless, the MHE-based method achieves significantly lower drift levels.

Figure 6b presents the position estimation results under external disturbances, with the perturbed periods highlighted in pink. Compared to the undisturbed case, drift is more pronounced, but the proposed method still maintains higher accuracy and stronger robustness against disturbances.

**Figure 7:**
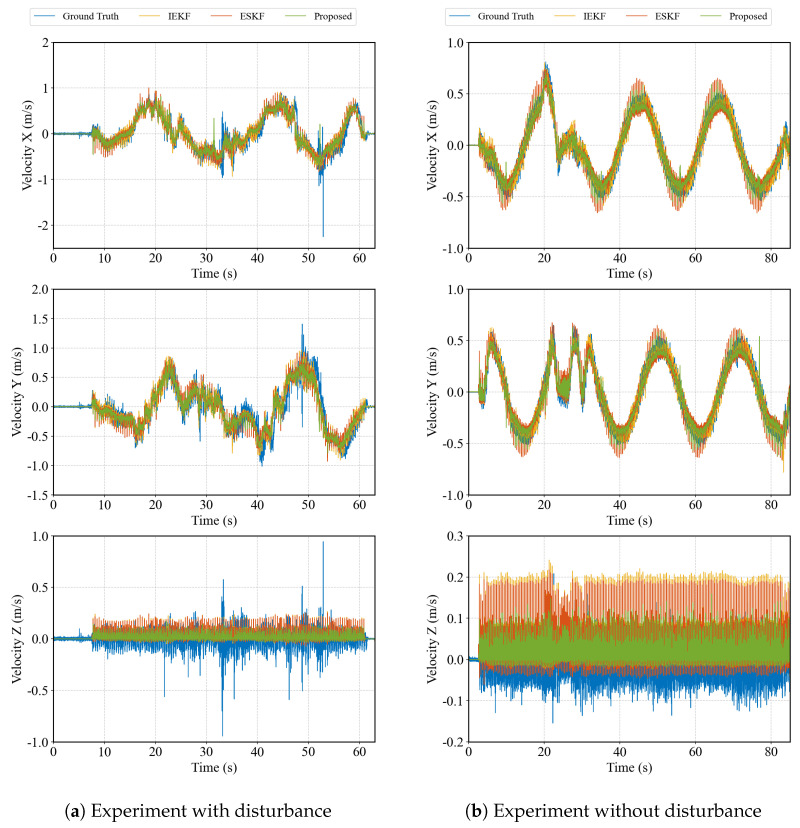
The comparison of absolute positions in the space.

Figure 7 compares the velocity estimation in three directions. As Vicon cannot directly measure velocity, ground truth was obtained by differentiating position data. Under disturbance conditions, the ground truth exhibits substantial noise. All three estimators effectively track velocity and filter noise, with the proposed method showing significantly lower noise levels.

**Figure 8:**
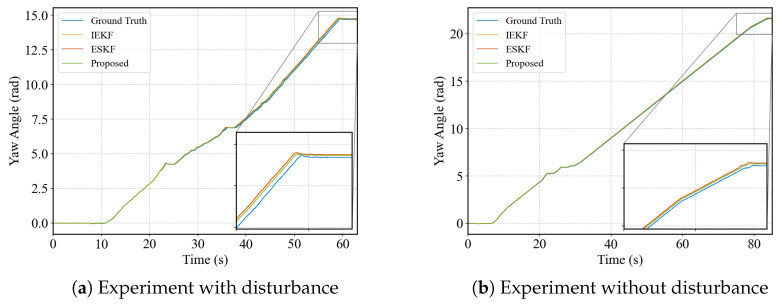
The comparison of absolute positions in the space.

Figure 8 compares rotational estimation. Vicon represents orientation using quaternions, while our estimator is based on Lie group representations. For clearer comparison and interpretation, all results were converted into Euler angles. Since the experiments were performed on flat terrain, the roll and pitch angles remained nearly constant and are omitted. At the beginning of the motion, both estimators closely match the ground truth yaw angle. However, with extended operation, drift becomes inevitable, although the proposed method exhibits slightly lower drift.

**Figure 9:**
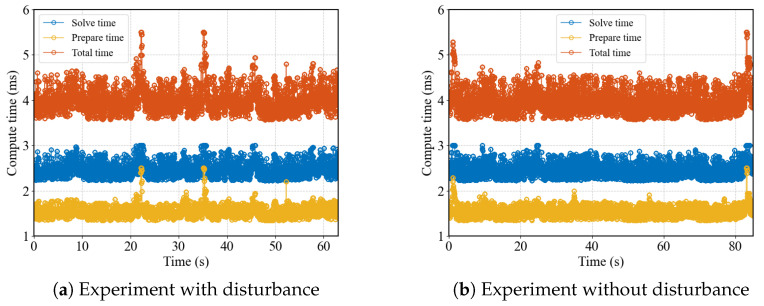
The comparison of absolute positions in the space.

Figure 9 shows the computation time. With a rolling horizon of 25 steps, matrix preparation takes approximately 1.5 ms. The quadratic programming solver used is Proxsuite, with a typical solving time of around 2.5 milliseconds. To maintain a stable update rate for the state estimator, the computation is forcibly terminated if it exceeds 3 ms. In the original RTI method, the period is the sum of the preparation time and the solving time. In para-RTI, the period only depends on the solving time.

Table 1 presents the RMSE values of position, velocity, and orientation for the three methods across two different experiments. It can be seen that the proposed method achieves the lowest RMSE values in all cases.

## 6. Discussion

This paper presents a real-time proprioceptive state estimation framework for rigid body dynamics, formulated in the right trivialized tangent space of the SE(3) group using the Cayley transform. By integrating historical observation data with prior estimates from the ESKF, a receding horizon estimator is constructed, and the Para-RTI method is employed to efficiently update the nonlinear state estimates. Experimental results on a quadruped robot demonstrate that the proposed estimator effectively mitigates impact noise from ground contact and significantly reduces estimation drift. Furthermore, the framework exhibits strong potential for fusing multiple sensor inputs and incorporating physical constraints, providing a solid foundation for future research.

Nevertheless, long-term drift remains an inherent limitation of proprioceptive state estimation, primarily due to IMU integration errors and the cumulative effects of minor slippages during foot–ground impacts. The proposed method offers stability in scenarios where external sensing is limited, enabling the robot to maintain autonomous perception even in challenging environments. However, all current experiments are conducted indoors within a fixed area due to motion capture system constraints. In future work, we will further validate the effectiveness of the proposed approach in outdoor environments and incorporate neural network-based terrain estimation algorithms to enhance the robustness of the system. Overall, the results indicate that the proposed framework is a promising solution for robust and accurate state estimation in legged robots, especially under conditions with limited external sensing.

## Figures and Tables

**Table 1:** RMSE comparison.

	Proposed	ESKF	IEKF
Position RMSE w/o disturbance (m)	0.0292	0.0472	0.0539
Velocity RMSE w/o disturbance (m/s)	0.0565	0.0592	0.0594
Angle RMSE w/o disturbance (rad)	0.0376	0.0684	0.0687
Position RMSE w/ disturbance (m)	0.1278	0.1601	0.1841
Velocity RMSE w/ disturbance (m/s)	0.1232	0.1268	0.1262
Angle RMSE w/ disturbance (rad)	0.0677	0.1165	0.1085

## Data Availability

The original contributions presented in this study are included in the article. Further inquiries can be directed to the corresponding author.

## References

[1] Du W., Fnadi M., Benamar F. (2020). Rolling based locomotion on rough terrain for a wheeled quadruped using centroidal dynamics. Mech. Mach. Theory.

[2] Xu K., Wang S., Shi L., Li J., Yue B. (2025). Horizon-stability control for wheel-legged robot driving over unknow, rough terrain. Mech. Mach. Theory.

[3] Chen L., Ye S., Sun C., Zhang A., Deng G., Liao T., Sun J. CNNs based foothold selection for energy-efficient quadruped locomotion over rough terrains. Proceedings of the 2019 IEEE International Conference on Robotics and Biomimetics (ROBIO).

[4] Sola J. (2017). Quaternion kinematics for the error-state Kalman filter. arXiv.

[5] Hartley R., Ghaffari M., Eustice R.M., Grizzle J.W. (2020). Contact-Aided Invariant Extended Kalman Filtering for Robot State Estimation. Int. J. Robot. Res..

[6] Li J., Yuan Z., Sang X., Zhou Q., Zhang J. (2023). Center of Mass Dynamics and Contact-Aided Invariant Filtering for Biped Robot State Estimation. IEEE Sens. J..

[7] Wisth D., Camurri M., Fallon M. (2023). VILENS: Visual, Inertial, Lidar, and Leg Odometry for All-Terrain Legged Robots. IEEE Trans. Robot..

[8] Kim Y., Yu B., Lee E.M., Kim J.h., Park H.w., Myung H. (2022). STEP: State estimator for legged robots using a preintegrated foot velocity factor. IEEE Robot. Autom. Lett..

[9] Kang J., Kim H., Kim K.S. (2023). VIEW: Visual-Inertial External Wrench Estimator for Legged Robot. IEEE Robot. Autom. Lett..

[10] Yang S., Zhang Z., Bokser B., Manchester Z. Multi-IMU Proprioceptive Odometry for Legged Robots. Proceedings of the 2023 IEEE/RSJ International Conference on Intelligent Robots and Systems (IROS).

[11] Vukov M., Gros S., Horn G., Frison G., Geebelen K., Jørgensen J., Swevers J., Diehl M. (2015). Real-Time Nonlinear MPC and MHE for a Large-Scale Mechatronic Application. Control Eng. Pract..

[12] López-Negrete R., Biegler L.T. (2012). A moving horizon estimator for processes with multi-rate measurements: A nonlinear programming sensitivity approach. J. Process Control.

[13] Diehl M., Ferreau H.J., Haverbeke N., Morari M., Thoma M., Magni L., Raimondo D.M., Allgöwer F. (2009). Efficient Numerical Methods for Nonlinear MPC and Moving Horizon Estimation. Nonlinear Model Predictive Control.

[14] Khorshidi S., Dawood M., Bennewitz M. (2024). Centroidal State Estimation based on the Koopman Embedding for Dynamic Legged Locomotion. arXiv.

[15] Kang J., Wang Y., Xiong X. (2024). Fast Decentralized State Estimation for Legged Robot Locomotion via EKF and MHE. arXiv.

[16] Sola J., Deray J., Atchuthan D. (2018). A micro Lie theory for state estimation in robotics. arXiv.

[17] Mangiacapra G., Wittal M., Capello E., Nazari M. (2022). Unscented Kalman Filter and Control on TSE(3) with Application to Spacecraft Dynamics. Nonlinear Dyn..

[18] Teng S., Chen D., Clark W., Ghaffari M. (2023). An Error-State Model Predictive Control on Connected Matrix Lie Groups for Legged Robot Control. arXiv.

[19] Agrawal A., Chen S., Rai A., Sreenath K. (2022). Vision-Aided Dynamic Quadrupedal Locomotion on Discrete Terrain Using Motion Libraries. arXiv.

[20] Müller A. (2021). Review of the exponential and Cayley map on SE (3) as relevant for Lie group integration of the generalized Poisson equation and flexible multibody systems. Proc. R. Soc. A.

[21] Torrisi G., Grammatico S., Smith R.S., Morari M. A Variant to Sequential Quadratic Programming for Nonlinear Model Predictive Control. Proceedings of the 2016 IEEE 55th Conference on Decision and Control (CDC).

[22] Verschueren R. (2018). Convex Approximation Methods for Nonlinear Model Predictive Control. Doctoral Dissertation.

[23] Gros S., Zanon M., Quirynen R., Bemporad A., Diehl M. (2020). From Linear to Nonlinear MPC: Bridging the Gap via the Real-Time Iteration. Int. J. Control.

[24] Hespanhol P., Quirynen R. A Real-Time Iteration Scheme with Quasi-Newton Jacobian Updates for Nonlinear Model Predictive Control. Proceedings of the 2018 European Control Conference (ECC).

[25] Nurkanović A., Zanelli A., Albrecht S., Frison G., Diehl M. (2020). Contraction Properties of the Advanced Step Real-Time Iteration for NMPC. IFAC-PapersOnLine.

